# The Ubiquitin E3 Ligase MaLUL2 Is Involved in High Temperature-Induced Green Ripening in Banana Fruit

**DOI:** 10.3390/ijms21249386

**Published:** 2020-12-09

**Authors:** Wei Wei, Jian-ye Chen, Ze-xiang Zeng, Jian-fei Kuang, Wang-jin Lu, Wei Shan

**Affiliations:** 1 State Key Laboratory for Conservation and Utilization of Subtropical Agro-bioresources/GuangdongProvincial Key Laboratory of Postharvest Science of Fruits and Vegetables/Engineering Research Center of Southern Horticultural Products Preservation, Ministry of Education, College of Horticulture, South China Agricultural University, Guangzhou 510642, China; weiwei_11663@163.com (W.W.); chenjianye@scau.edu.cn (J.-y.C.); 13760896634@163.com (Z.-x.Z.); jfkuang@scau.edu.cn (J.-f.K.); wjlu@scau.edu.cn (W.-j.L.); 2 Lingnan Guangdong Laboratory of Modern Agriculture, Guangzhou 510642, China

**Keywords:** banana fruit, chlorophyll degradation, E3 ubiquitin ligase, high temperature, green ripening

## Abstract

Harvested banana fruit ripened under warm temperatures above 24 °C remain green peel, leading to severe economic loss. E3 ubiquitin-ligases, as the major components in the ubiquitination pathway, have been implicated to play important roles in temperature-stress responses. However, the molecular mechanism underlying high temperature-triggered stay-green ripening bananas in association with E3 ubiquitin-ligases, remains largely unknown. In this study, a RING-type E3 ubiquitin ligase termed *MaLUL2*, was isolated and characterized from banana fruit. The *MaLUL2* gene contains 1095 nucleotides and encodes a protein with 365 amino acids. The MaLUL2 protein contains a domain associated with RING2 (DAR2) and a RING domain, which are the typical characteristics of RING-type E3 ligases. *MaLUL2* expression was up-regulated during high temperature-induced green ripening. Subcellular localization showed that MaLUL2 localized in the nucleus, cytoplasm, and plasma membrane. MaLUL2 displayed E3 ubiquitin ligase activity in vitro. More importantly, transient overexpression of *MaLUL2* in banana fruit peel increased the level of ubiquitination in vivo and led to a stay-green phenotype, accompanying with decreased expression of chlorophyll catabolic genes. Collectively, these findings suggest that MaLUL2 might act as a negative regulator of chlorophyll degradation and provide novel insights into the regulatory mechanism of high temperature-induced green ripening bananas.

## 1. Introduction

Banana (*Musa acuminata*) is a tropical fruit that belong to the *Musaceae* family, presenting as one of mostly popular fruits worldwide [1,2]. As a typical climacteric fruit, bananas are usually harvested at a green mature stage and transported to the wholesale markets, where they are treated with ethylene gas/ethephon to ripen before marketing [3]. The color of the banana peel turns rapidly from green to fully yellow, which is the most visible symptom during the fruit ripening process. Bananas usually ripen under ambient conditions at 16–24 °C. However, banana fruit could not exhibit a fully yellow peel and stay green when ripening under the temperatures above 24 °C due to the inhibition of chlorophyll degradation [4]. These green ripening fruits are regarded as poor quality by consumers and lead to a lower market price than golden yellow ripening bananas, causing a significant loss in economic value. Hence, the exploration of underlying mechanisms causing the stay-green ripening bananas will be of great practical value to maintain banana fruit quality under a high temperature.

Previously, several studies employing transcriptomic and proteomic technologies identified a host of structural and regulatory genes/proteins in bananas in response to a high temperature, among which genes/proteins related to chlorophyll degradation are mainly characterized [4]. For example, the expression level of stay-green protein (SGR) was indicated as the possible upstream regulator of chlorophyll degradation [5]. It was further proposed that high temperature-induced accumulation of soluble sugars in the peel could be the major factor inhibiting chlorophyll degradation, causing the green ripening bananas [5]. Genes/proteins involved in reactive oxygen species (ROS) scavenging, stress response, cell wall, and amino acid metabolism were also suggested to be associated with high temperature-induced green ripening [6]. Different from the stay-green ripening at a high temperature, elevated CO_2_-caused green ripening bananas at 20 °C were related to the inhibition of senescence and enhancement of anaerobic respiration [7]. Overall, stay-green ripening in banana fruit is a complicated process modulated by multiple factors, and especially the crucial regulatory proteins remain elusive.

Generally, mutated and damaged/misfolded proteins were produced when plants are encountered to environmental stresses including high temperature [8]. Subsequently, these proteins could be removed by the ubiquitin 26S proteasome system (UPS). Importantly, the stability of many regulatory proteins such as transcription factors is also controlled by UPS [9,10]. Accordingly, protein ubiquitination is considered as a versatile post-translational modification that plays important roles in mediating the accurate changes required for growth and development as well as adaption to abiotic and biotic stresses [11,12,13]. Noticeably, the diversity and recognition of substrate protein for ubiquitination is commonly controlled by the E3 ubiquitin ligases [14]. Thus, the identification of E3 ubiquitin ligases is extensively investigated. According to the reaction mechanism and special conserved domains, plant E3 ubiquitin ligases are divided into three types: HECT (Homology to E6-Associated Carboxy-Terminus), RING (Really Interesting New Gene), and U-box [15,16]. Among these ubiquitin ligases, RING-type E3 ligases are widely studied, and have been revealed to participate in plant growth and development, as well as in stress responses such as temperature stresses [17,18]. For instance, an Arabidopsis RING-type E3 ligase AtATL78 was up-regulated by cold stress, and acted as a negative regulator of cold stress response [19]. In banana, a RING-type E3 ligase MaSINA1 might negatively regulate the cold stress response of fruit [20]. Similarly, a RING-type E3 ubiquitin ligase MdMIEL1 was found to negatively modulate cold tolerance and anthocyanin accumulation in apple fruit [21]. In addition, several RING-type E3 ligases in rice including HCI1, HTAS, and HIRP1 are reported to be involved in heat stress tolerance [8]. UPS has been implicated to have causative roles in stay-green during senescence [22,23,24], and the regulation of chloroplast-targeted pre-proteins [25]. Nevertheless, the mechanistic basis as to how the stay-green in fruit is modulated by E3 ubiquitin ligases remains largely unknown.

By screening our RNA-seq transcriptome database, a banana fruit E3 ubiquitin ligase termed MaLUL2 was found to be clearly up-regulated by high temperature, raising the possibility that MaLUL2 is involved in modulating stay-green ripening. To reveal the regulatory mechanism of MaLUL2, in this study, we further characterized its expression pattern in relation to high temperature-induced green ripening. MaLUL2 possessed E3 ligase activity. Moreover, transient overexpression of *MaLUL2* in banana fruit led to a stay-green phenotype by affecting chlorophyll catabolism. These data provide novel insights into the regulatory mechanism of high temperature-induced green ripening bananas.

## 2. Results

### 2.1. High Temperature Induces Stay-Green Ripening in Banana Fruit

**Figure 1:**
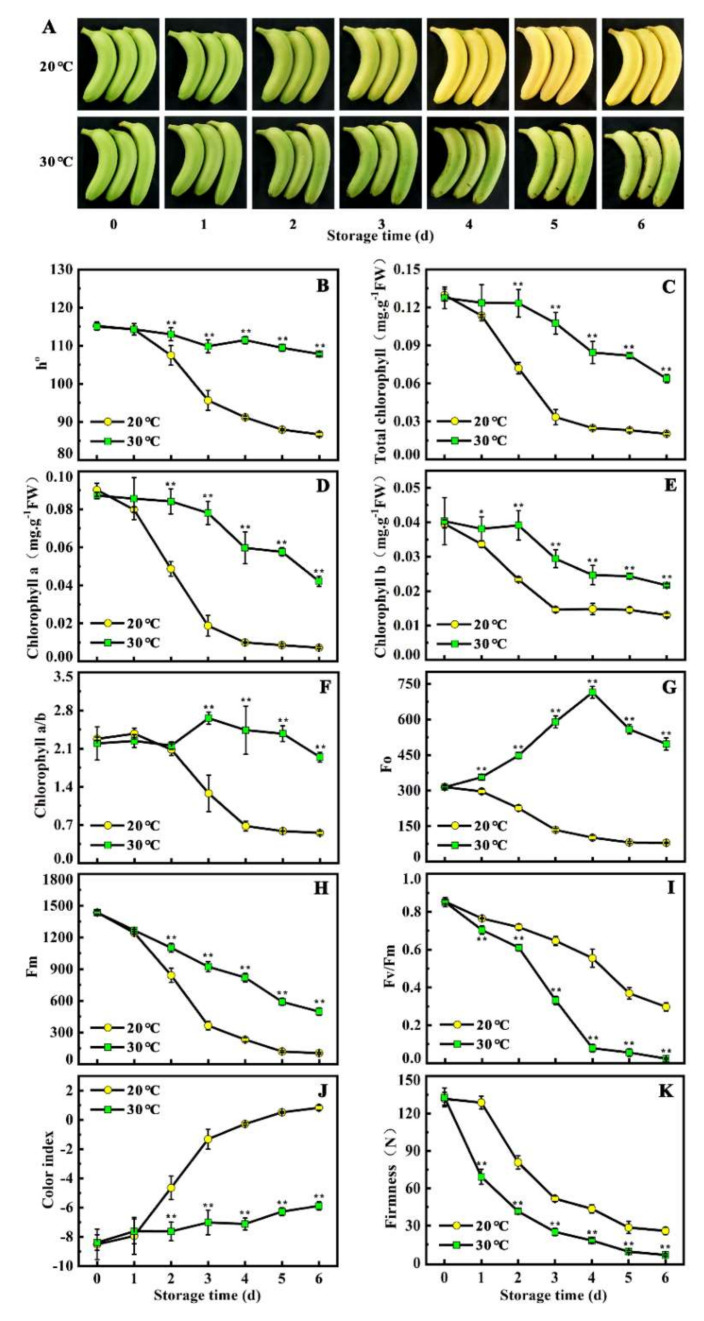
High temperature induces stay-green ripening in banana fruit. (**A**) Appearance of ripening fruit stored at 20 °C (control) and 30 °C (high temperature). (**B**–**K**) Changes of h°, total chlorophyll content, chlorophyll a, chlorophyll b, chlorophyll a/b, Fo, Fv, Fm, Fv/Fm, color index, and fruit firmness in fruit stored at 20 °C and 30 °C during ripening. Fruit is pre-treated with 100 μL/L ethylene for 18 h at 20 °C, and, subsequently, stored at 20 °C and 30 °C for six days, respectively. Data are the mean ± S.E. of three biological replicates. Asterisks indicate significant differences by a student’s *t*-test (* *p* < 0.05, ** *p* < 0.01).

To assess the effects of temperature on ripening and peel color in banana fruit, h° (hue angle), total chlorophyll content, chlorophyll a, chlorophyll b, chlorophyll a/b, chlorophyll fluorescence parameters (Fo, Fv, Fm, and Fv/Fm), color index, and fruit firmness during the six days of storage were evaluated. As shown in Figure 1A, during the storage at 20 °C, banana fruit began to turn yellow after 2 days of ethylene treatment and completely de-greened after 5 days. However, no clear yellowing phenomenon was observed when bananas ripened under 30 °C (high temperature), and fruits turned out to be stay-green ripening (Figure 1A). Consistently, h° and total chlorophyll content in the fruit under 30 °C were higher, maintaining 1.24-fold and 4.34-fold at 6 days, respectively, compared with levels in the fruit under 20 °C (Figure 1B,C). Similar trends were observed for the content of chlorophyll a and chlorophyll b, chlorophyll a/b, Fo, Fv, and Fm, which were maintained in higher levels in the fruit under 30 °C than that of the fruit under 20 °C (D–H). On the contrary, Fv/Fm, color index, and fruit firmness in the fruit under 30 °C were lower, when compared with the levels in the fruit under 20 °C (Figure 1 I–K). These results indicate that banana fruit stored at 30 °C inhibits chlorophyll degradation, thereby leading to stay-green ripening.

### 2.2. Identification of Banana Fruit MaLUL2

**Figure 2:**
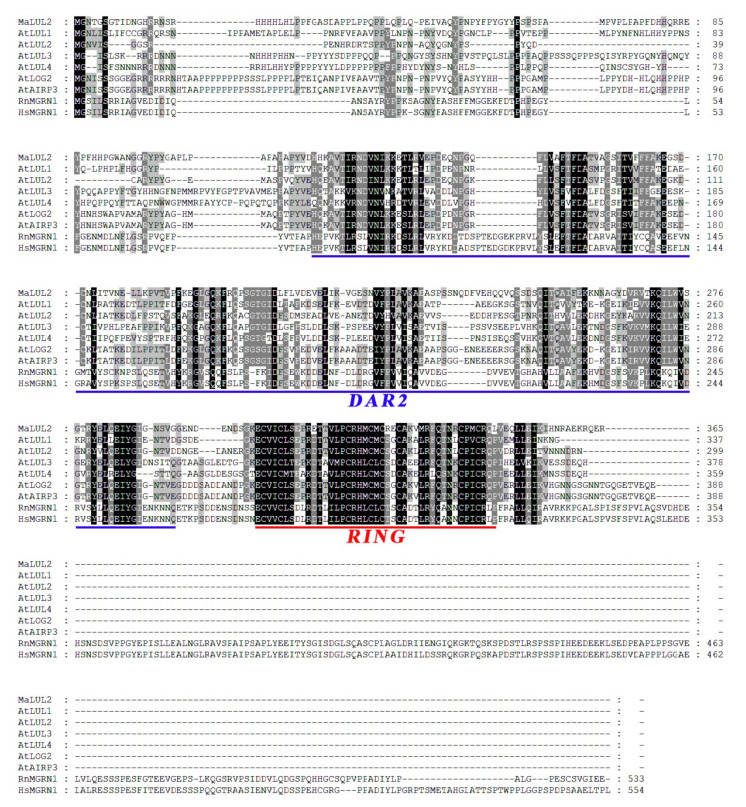
Multiple sequence alignment of MaLUL2. Identical and similar amino acids were shaded in black and grey, respectively. The DAR2 domain and RING motif are underlined with blue and red, respectively. The following proteins were used for analysis: AtLUL1 (NP_195940.1), AtLUL2 (NP_190909.1), AtLUL3 (NP_197409.1), AtLUL4 (NP_566274.1), AtLOG2 (NP_566356.1), AtAIRP3 (NP_566356.1), RnMGRN1 (NP_001013986.1), and HsMGRN1 (NP_001135761.2).

To better understand the E3 ubiquitin ligases medicated-protein ubiquitination in association with fruit quality affected by high temperature, differential expression of genes encoding E3 ubiquitin ligases were screened in our RNA-seq transcriptome database associated with high temperature-induced green ripening bananas. An E3 ubiquitin ligase that was clearly up-regulated by high temperature attracted our attention. The homology search showed that this E3 ubiquitin ligase shared 47% similarity with LOSS OF GDU2 (LOG2) −LIKE UBIQUITIN LIGASE2 (AtLUL2, At3g53410) at the amino acid level, which is identified as the closest homologue to AtLUL2. Thus, this E3 ubiquitin ligase was designated as MaLUL2 (GSMUA_Achr11G00510_001). MaLUL2 contains an open reading frame (ORF) of 1095 bp that encodes a putative protein of 365 amino acids with a predicted molecular weight of 40.91 kDa and calculated *p*I of 6.31. Sequence alignment of MaLUL2 with other plants LOG2−LIKE UBIQUITIN LIGASEs (LULs) displays the presence of the DAR2 domain in the central region and a RING motif in the C-terminal region, which are the typical characteristics of RING−type E3 ligases (Figure 2).

### 2.3. MaLUL2 is Up-Regulated Under a High Temperature

**Figure 3:**
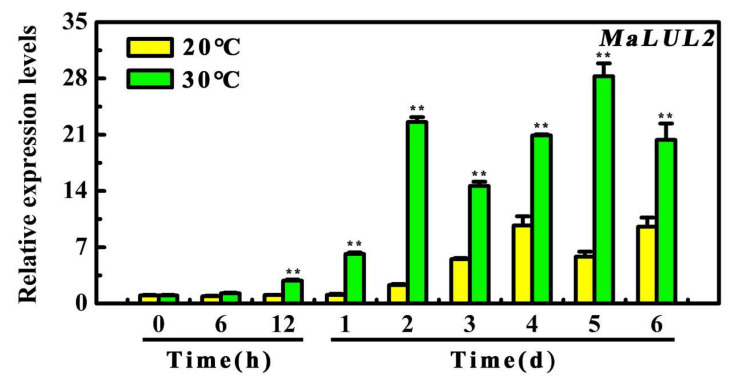
Relative expression of *MaLUL2* in fruit stored at 20 °C and 30 °C during ripening. Each value represents the mean ± S.E. of three biological replicates. Asterisks indicate significant differences by student’s *t*-test (** *p* < 0.01).

To further explore the possible association of MaLUL2 with high temperature-induced green ripening bananas, its expression patterns during the 6 days of storage under 20 °C and 30 °C were examined by quantitative real-time PCR (qRT-PCR). As shown in Figure 3, the transcript level of MaLUL2 significantly increased in fruit under 30 °C throughout the whole storage period with a 9.8-fold and 5.85-fold higher than those of fruit under 20 °C on days 2 and 5, respectively. The result that high temperature made a marked induction in the expression of MaLUL2 indicates the possible involvement of MaLUL2 in a stay-green ripening process.

### 2.4. Sub-Cellular Localization and In Vitro Self-Ubiquitination of MaLUL2

**Figure 4:**
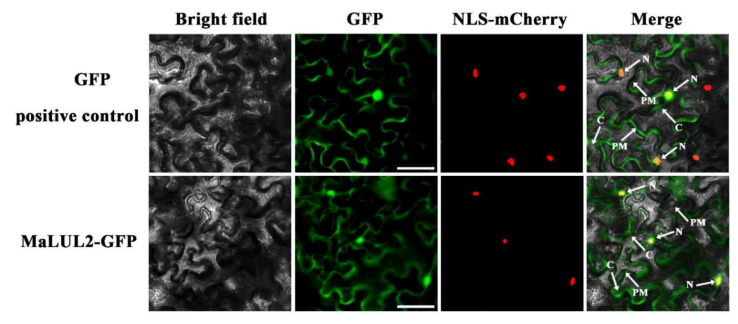
Subcellular localization of MaLUL2 in epidermal cells of *Nicotiana benthamiana* leaves. A plasmid harboring GFP or MaLUL2−GFP was transformed into *Nicotiana benthamiana* leaves by *Agrobacterium tumefaciens* strain EHA105. GFP signals were observed with a fluorescence microscope after 2 days of infiltration. The NLS−mCherry was included in each transfection to serve as a control for successful transfection as well as for nuclear localization. Cytoplasm (C), plasma membrane (PM), and nucleus (N) are indicated by arrows. Bars, 50 μm.

**Figure 5:**
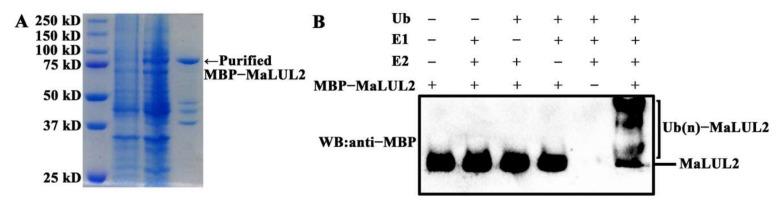
MaLUL2 protein exhibits E3 ubiquitin ligase activity. (**A**) The SDS-PAGE gel stained with Coomassie brilliant blue demonstrating affinity purification of the recombinant MBP−MaLUL2 protein used for a ubiquitination assay. (**B**) The in vitro ubiquitination assay showing the E3 ubiquitin ligase activity of MaLUL2. Purified recombinant MBP−MaLUL2 protein was incubated with Ub, human E1, and human E2 UbcH5b at 30 °C for 2 h. The reaction mixture was analyzed by immunoblotting with an anti−Maltose-Binding Protein (MBP) antibody. Ubiquitination results in a heterogeneous collection of higher molecular mass proteins that were detected using an anti−MBP antibody.

To visualize the sub-cellular localization of MaLUL2, it was fused with the green fluorescent protein (GFP) and transient expressed in tobacco leaf epidermal cells. Similar with the fluorescence of control 35S−GFP distributed throughout of the cell, the GFP fluorescence of MaLUL2 fusion protein was detected in the nucleus, cytoplasm, and plasma membrane (Figure 4). Several studies have shown that proteins with a RING domain exhibit E3 ubiquitin ligase activities [26,27]. Since MaLUL2 belonged to RING-type E3 ligases, we sought to determine whether MaLUL2 harbors E3 ubiquitin ligase activity in vitro. MaLUL2 tagged with the maltose binding protein (MBP) was expressed in *E.coli* and an in vitro ubiquitination assay was subsequently conducted using a purified MBP−MalUL2 protein (Figure 5A). As shown in Figure 5B, poly-ubiquitinated chains could be formed when MaLUL2 was incubated with human E1, human E2, and the substrate ubiquitin (Ub). In the absence of any of the reaction components, no poly-ubiquitinated chains were observed. These data clearly suggest that the MaLUL2 protein possesses E3 ubiquitin ligase activity.

### 2.5. Transient Overexpression of MaLUL2 in Banana Peel Inhibits Chlorophyll Degradation

**Figure 6:**
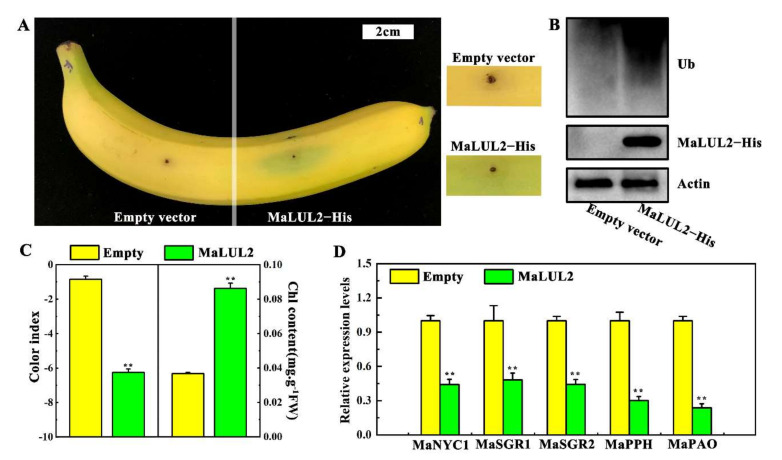
Effect of transient overexpression of *MaLUL2* in banana fruit peel on chlorophyll degradation. (**A**) Appearance of banana fruit transiently overexpressing *MaLUL2* and empty vector (control). (**B**) Immunoblotting analysis showing the level of ubiquitination and MaLUL2 protein in *MaLUL2*−overexpressing and control banana fruit peel. (**C**) Changes of the color index and total chlorophyll content in *MaLUL2*−overexpressing and control banana fruit peel. (**D**) Relative mRNA abundance of chlorophyll catabolic genes including *MaNYC1*, *MaSGR1*, *MaSGR2*, *MaPPH*, and *MaPAO* in *MaLUL2*−overexpressing and control banana fruit peel. Three days after ethylene treatment at 20 °C, the injection peel of *MaLUL2*−overexpressing and control fruit were sampled for assays. Data presented in C and D are means ± S.E. of three independent biological replicates. Asterisks indicate significant differences by student’s *t*-test (** *p* < 0.01).

The possible biological function of MaLUL2 in stay-green ripening was verified through transient overexpression in a banana peel. Compared with the empty vector, transient overexpression of *MaLUL2* in fruit peel led to a stay-green phenotype near the injection point within 3 days after ethylene treatment at 20 °C (Figure 6A). Moreover, immunoblotting analysis demonstrated that overexpression of *MaLUL2* significantly enhanced the levels of ubiquitination in the peel (Figure 6B). Concomitantly, a lower color index and higher chlorophyll content were found in a *MaLUL2* injected section than those in an empty injected section (Figure 6C). Additionally, the expressions of banana chlorophyll catabolic genes including *MaNYC1*, *MaSGR1*, *MaSGR2*, *MaPPH*, and *MaPAO* were down-regulated in a *MaLUL2*−overexpressing fruit peel (Figure 6D). These data indicate that transient overexpression of *MaLUL2* in banana peel inhibits chlorophyll degradation, thereby leading to stay-green ripening.

## 3. Discussion

With global warming, the storage temperature of horticultural fruits and vegetables after picking rises gradually in the natural environment, reducing their commercial quality and shortening their shelf-life. For instance, harvested bananas that ripen under high temperature display stay-green fruit peel, which affects the appearance quality and causes economic loss [4,5]. High temperature is a common abiotic stress that adversely affects the plant biological process by the accumulation of various abnormal proteins. The UPS serves as a versatile post-translational modification, and has been implicated to play critical roles in responses to biotic and abiotic stresses in plants [8,9,13]. E3 ubiquitin ligases are crucial components of UPS [14,15]. In this study, we identified and characterized a RING E3 ubiquitin ligase MaLUL2 from banana fruit, and provided evidence to support the possible involvement of MaLUL2 in high temperature-induced green ripening in banana fruit.

Heat stress is one of the major abiotic stresses that affects plant growth, development, and crop yield. Up until now, several E3 ubiquitin ligases have been reported to be responsive to heat stress. In Arabidopsis, the transcription of U-box type ubiquitin E3 ligase *AtCHIP* and *AtPUB48* are rapidly induced in seeds after high temperature treatment [28,29]. In addition, expression of RING type E3 ligases *OsHCI1*, *OsHTAS*, and *OsHIRP1* from rice are also up-regulated under heat stress treatment at the seedling stage [30,31,32]. Similarly, we found that the RING-type E3 ligase gene *MaLUL2* was up-regulated when high temperature caused green ripening in banana fruit (Figure 1 and Figure 3). This illustrates that the MaLUL2 may be involved in a high temperature response and development of green ripening bananas.

E3 ubiquitin ligases regulate heat stress responses in several ways. Overexpression of *AtPUB48* enhances heat stress-inducible genes such as *HSP101*, *HSP70*, *HSP25.3*, *HSFA2*, and *ZAT12* to confer heat stress tolerance in transgenic Arabidopsis [29]. OsHTAS promotes hydrogen peroxide accumulation and leads to stomatal closure in rice leaves, thus, enhancing heat tolerance [31]. Another rice RING finger E3 ligase OsHCI1 enhances acquired thermotolerance by modulating nuclear–cytoplasmic trafficking of nuclear substrate proteins via mono-ubiquitination [30]. However, the association of E3 ubiquitin ligases with quality deterioration in fruits under high temperature is less reported. Recently, an E3 ubiquitin ligase MdMIEL1 was found to negatively regulate cold tolerance and anthocyanin accumulation in apple fruit by degrading MdMYB308L protein and then inhibiting MdMYB308L-activated expressions of *MdCBF2* and *MdDFR* [21]. Previous studies have suggested that the green ripening of bananas is mainly due to the suppression of chlorophyll breakdown under high temperature [5,33,34]. In this study, transient overexpression of *MaLUL2* in banana peel lead to a stay-green phenotype and decreased expression of chlorophyll catabolic genes, suggesting that *MaLUL2* acts as a negative regulator of chlorophyll degradation in the development of green ripening bananas. Based on previous reports and the results of the present study, E3 ubiquitin ligases may play an important role in pigment metabolism under extreme temperatures in fruits. We also found that the inhibition of chlorophyll b by high temperature was stronger than that of chlorophyll a (Figure 1D–F). It has been shown that chlorophyll b is essential for the proper assembly of PSII, whereas the degradation of PSII core protein D1 promotes PSII inactivation [35,36,37]. Thus, it will be interesting to verify whether D1 protein degradation is associated with green ripening of bananas.

Proteomic analysis showed large numbers of proteins were differentially expressed between the normal and green ripening bananas, indicating that high temperature influences banana fruit ripening at the proteomic level [4,6]. E3 ubiquitin ligases specifically recognize substrates and mediate their ubiquitination and degradation, which is a well-established pathway to balance protein levels [8]. Our recent study in bananas showed that a RING E3 ligase MaXB3 acts as a negative regulator of ethylene biosynthesis during banana fruit ripening by degrading ethylene biosynthesis enzyme MaACS1 and MaACO1 and ripening-related transcription factor MaNAC2 [27]. Here, we found that the MaLUL2 protein has a DAR2 domain (Figure 2), which is reported to be responsible for targeting substrates [38]. The in vitro ubiquitination assay demonstrated that MaLUL2 had E3 ubiquitin ligase activity (Figure 5). Furthermore, transient overexpression of MaLUL2 in banana peel increased the level of ubiquitination in vivo (Figure 6B), and inhibited chlorophyll degradation by down-regulating expression of chlorophyll catabolic genes. These results indicate that MaLUL2 has the ability to mediate the ubiquitination of substrate proteins. However, the involvement of MaLUL2 in the development of stay-green ripening bananas by directly targeting chlorophyll degradation-related proteins for degradation via the UPS needs to be further elucidated.

## 4. Materials and Methods

### 4.1. Plant Materials and Samples

Pre-climacteric banana (*Musa acuminate*, AAA group, cv. Cavendish) fruits at 70–80% maturation were obtained from a local commercial plantation near Guangzhou, South-eastern China. Each banana hand was separated into individual fingers, and fruits with a uniform shape, weight, and maturity as well as free of visual defects were used for this study. The fruits were immersed in 0.05% (*w*/*v*) Sporgon for 3 min to prevent fungal disease. Ripening was initiated by treating the fruit with 100 μL/L ethylene in an airtight container for 18 h at 20 °C. After ripening initiation, fruits were allowed to store at 20 °C and 30 °C (high temperature) in unsealed polyethylene plastic bags for six days, respectively. Three fruits were picked from each group and subjected to the measurement of color, chlorophyll content, and firmness every day. The sampled peel tissues were also collected, frozen in liquid nitrogen, and stored at −80 °C immediately for further analysis.

### 4.2. Assessment of Ripening Parameters

Fruit firmness was determined using a penetrometer (model no. 5542, Instron) with a 1-cm diameter probe mounted on a motorized test stand. The firmness was recorded as the maximum peak force in Newton (N) achieved during compression and extrusion. Peel color was measured by diffuse reflectance using a chroma meter. The chroma meter allocates color coordinates to each sample using the 3-dimensional L* × a* × b* color space, and the readings were calculated as h° (hue angle), L*, a* and b*. h° value is defined as a color wheel, with a red-purple color at an angle of 0°, yellow color at 90°, bluish-green color at 180°, and blue color at 270°. The L* represents lightness, the a* denotes redness (+)/greenness (−) and the b* displays yellowness (+)/blueness (−). The color index was further calculated by following the formula: 1000 × a*/L* × b*. Total chlorophyll content was measured as previously described [39]. Briefly, 2 g of banana peel was extracted in 25 mL of 80% acetone in the dark for 36 h and the absorbance of extracts at 663 nm and 645 nm were subsequently measured, respectively, using a spectrophotometer. Chlorophyll fluorescence was calculated noninvasively using Imaging-PAM-M series chlorophyll fluorometer (Heinz Walz) in the ‘Fv/Fm’ mode. Fo (minimum fluorescence), Fv (variable fluorescence), Fm (maximum fluorescence), and Fv/Fm (exciton transfer efficiency) were determined after dark adaptation for 30 min at room temperature as described previously [5,7].

### 4.3. RNA Extraction, Gene Cloning, and Sequence Analysis

Total RNA from frozen samples was extracted according to the hot borate method [40]. The cDNA was generated using HiScript II Q RT SuperMix for the qPCR (+gDNA wiper) kit (Vazyme Biotech, Nanjing, China). The full-length of *MaLUL2* (GSMUA_Achr11G00510_001) was isolated from our transcriptome database and blasted in NCBI (XM_009383691.2). Theoretical isoelectric points (*p*I) and mass values were assessed on the website (http://web.expasy.org/compute_pi/). Sequence alignment of LUL proteins were carried out with the CLUSTALW program (version 1.83).

### 4.4. Gene Expression Analysis by qRT-PCR

Quantitative real-time PCR (qRT-PCR) analysis was performed on a CFX96 (Bio-Rad Laboratories) using a SYBR Green PCR Master Mix (Promega), according to the following profile: 95 °C for 5 min, 40 cycles of 95 °C for 10 s, 60 °C for 30 s, and 72 °C for 30 s. *ACT1* was used as the reference gene to normalize the target gene expression levels [40].

### 4.5. Sub-Cellular Localization Assay

The coding sequence fragment of *MaLUL2* without the stop codon was amplified and inserted into the pEAQ− Green Fluorescent Protein (GFP) vector [41] to produce the fusion protein MaLUL2−GFP. The constructed plasmid and control vector pEAQ−GFP were transiently expressed in tobacco (*Nicotiana benthamiana*) leaf by *Agrobacterium*-mediated infiltration. *Agrobacteria* were collected by centrifugation at 1000× *g*, and re-suspended in MES buffer (10 mM MES, 10 mM MgCl_2_, 100 mM acetosyringone) to an absorbance of 0.3–0.5 at 600 nm, and incubated at room temperature for 3 h before infiltration. The suspensions were collected in a 1-mL syringe and carefully press-infiltrated manually into the leaves of 4-week-old tobaccos as described previously [42]. The NLS-mCherry was included in each transfection to serve as a control for successful transfection as well as for nuclear localization. Two days after the infiltration, GFP and mCherry signals were captured and photographed with a Zeiss fluorescence microscope.

### 4.6. Recombinant Protein Induction and Purification

For recombinant protein expression, the full-length cDNA of *MaLUL2* was amplified and cloned into the pMAL-c2X expression vector (New England Biolabs) with the maltose binding protein (MBP) tag. The recombinant MBP−MaLUL2 construct was expressed in *BM Rosetta* (DE3). When the transformed DE3 cell density reached OD_600_ = 0.6, the recombinant protein was induced by the addition of 0.5 mM isopropyl thio-β-D-galactoside at 28 °C for 6 h. Then the recombinant protein was purified by affinity chromatography using amylose resin (New England Biolabs), according to the manufacturer’s instructions. The purified protein was confirmed for size and purity by SDS-PAGE and Coomassie Brilliant Blue staining.

### 4.7. In Vitro Ubiquitination Assay

The in vitro ubiquitination assay was conducted as described previously [20,27]. Briefly, 500 ng of the MBP−MaLUL2 recombinant protein was incubated for 2 h in the presence or absence of 50 ng of human E1 (Boston Biochemistry), 250 ng of human E2 (Boston Biochemistry), and 2 mg of ubiquitin (Boston Biochemistry). The reaction products were visualized by a Western blot using anti-MBP (Abcam).

### 4.8. Transient Overexpression Analysis in Banana Fruit

The coding region of MaLUL2 fused with the 6 × His tag (GTGATGGTGATGGTGATG) was amplified and subcloned into a pCXUN vector under the control of a maize Ubiquitin promoter [43]). The recombinant MaLUL2−His construct and the vector control pCXUN were expressed in EHA105. The suspensions containing MaLUL2−His and the control pCXUN were press-infiltrated manually into the mature green banana peel, respectively, as described previously [27]. Transformed fruits were treated with 100 µL/L ethylene and stored at 20 °C. Samples were collected on day 4 after Agrobacterium introduction, for the measurement of the color index, chlorophyll content, gene expression, and protein accumulation.

### 4.9. Primers

All primers designed and used in this study are listed in Table S1.

## 5. Conclusions

Taken together, high temperature causes stay-green ripening in banana fruit. More importantly, a high temperature-induced RING-type E3 MaLUL2 is likely to act as a negative regulator of chlorophyll degradation, at least in part, by down-regulating expression of chlorophyll catabolic genes. These findings expand our understanding of E3 ligases’ functions and shed light on the regulatory mechanism of high temperature-induced green ripening bananas, and, thereby, lay the foundation for exploring new effective techniques to maintain the post-harvest quality of banana fruit. To the best of our knowledge, it is the first report of a heat stress-responsive E3 ubiquitin ligase in fruit.

## Supplementary Materials

Supplementary Materials can be found at https://www.mdpi.com/1422-0067/21/24/9386/s1, Table S1: Summary of primers used in this study.

## Abbreviations

GFP	Green fluorescent protein
HECT	Homology to E6-associated carboxy-terminus
MBP	Maltose binding protein
ORF	Open reading frame
qRT-PCR	Quantitative real-time PCR
ROS	Reactive oxygen species
SGR	Stay-green protein
UPS	Ubiquitin 26S proteasome system
