# Supplementary material for: The Ubiquitin E3 Ligase MaLUL2 Is Involved in High Temperature-Induced Green Ripening in Banana Fruit
Source: Int J Mol Sci. 2020 Dec 9;21(24):9386. doi: 10.3390/ijms21249386 (PMC7763436; doi:10.3390/ijms21249386)
Supplement: Supplementary file 1 [file ijms-21-09386-s001.pdf]

Supplementary Materials

Table S1. Summary of primers used in this study.

Assay	Primer sequence (5'-3')	Restriction Site	qRT-PCR product length
**Full length cloning**	*MaLUL2-F:* ATGGGGAACACGGGGAGCAG		
	MaLUL2-R: TCTCTCTTGTCGCTTCTCGGCT		
**Subcellular localization**	*MaLUL2-pEAQ-GFP-F:* TATTCTGCCCAAATTCGCGACCGGTATGGGGAACACGGGGAGCGGCACCAT	*Age* I	
	MaLUL2-pEAQ-GFP -R: AAAGTTCTTCTCCTTTGCTAGTCATTCTCTCTTGTCGCTTCTCGGCTCTGT	*Age* I	
**qRT-PCR**	*MaActin1-qF:* TGGTATGGAAGCCGCTGGTA		263bp
	MaActin1-qR: TCTGCTGGAATGTGCTGAGG		
	MaLUL2-qF: ACAGATTACACAGGCGATTTTTG		219bp
	MaLUL2-qR: TGTCTACAAGGCAGAACAGTGGT		
	MaNYC1-qF: ACTTTAGTGCCAAGGATGCG		99bp
	MaNYC1-qR: AAGCCAAGCAGTCACCAAAG		
	MaSGR1-qF: TGCTATGCTTCCGCTGCCTGTT		239bp
	MaSGR1-qR: TGTGGGAGATGGCGAGGGTG		
	MaSGR2-qF: GGTTGCGAGGCTATTTGGG		123bp
	MaSGR2-qR: TCGCTGTGGGTGAGGGTGTA		
	MaPPH-qF: TTCTTGCTCCGAGGGTGG		226bp
	MaPPH-qR: AACTCGCCAGCAAACTCG		
	MaPAO-qF: CCTTTACCTTCAACGGTCCTG		233bp
	MaPAO-qR: CGCAAAGCATAAGCCACAGC		
**Ubiquitination assay**	*MaLUL2*-pMAL-F: AGGGAAGGATTTCAGAATTCATGGGGAACACGGGGAGCGGCACCAT	*Bam*H I	
	MaLUL2-pMAL-R: AGGTCGACTCTAGAGGATCCCTATCTCTCTTGTCGCTTCTCGGCTCTGT	*Eco*R I	
**Transient overexpression**	*MaLUL2-His-F:* TTCTGCCCAAATTCGCGACCGGTATGGGGAACACGGGGAGCGGCACCAT	*Age* I	
	MaLUL2-His-R: TGATGGTGATGGTGATGCCCGGGTCTCTCTTGTCGCTTCTCGGCTCTGT	*Sma* I	
